# Management of salivary gland carcinomas - a review

**DOI:** 10.18632/oncotarget.13952

**Published:** 2016-12-15

**Authors:** Xiaoli Wang, Yijun Luo, Minghuan Li, Hongjiang Yan, Mingping Sun, Tingyong Fan

**Affiliations:** ^1^ School of Medical and Life Sciences, University of Jinan-Shandong Academy of Medical Sciences, Jinan, Shandong, China; ^2^ Department of Radiation Oncology, Shandong Cancer Hospital and Institute, Jinan, Shandong, China

**Keywords:** salivary gland cancers, adenoid cystic carcinoma, elective neck dissection, chemotherapy, targeted therapy

## Abstract

Salivary gland carcinomas are a heterogeneous group of tumors with many histological subtypes which occur in both major and minor salivary glands. However, they have a relatively low of incidence. Their rarity limits study size and the ability to perform phase III trials. Therefore, to date, the entire management is usually varied. Certain published studies have paid more attention to the systemic therapy in the management of metastatic or locally recurrent salivary gland cancer, while little effort has been made to study the entire management for this lesions. Although results of treatment for patients with salivary gland carcinoma have improved in recent years, the treatment of salivary gland cancers is still not standardized. And some patients who haven’t received optimal treatment strategies had a reduced survival. In this review, the topics covered include surgery and radiotherapy, selective neck dissection, chemotherapy, and targeted therapy, which aimed to summarize the optimal management approaches and to develop recommendations for managing this lesions. For these rare cancers, there is also a need for a determined, coordinated effort to conduct high-quality clinical trials.

## INTRODUCTION

Salivary gland cancers (SGCs) are relatively rare, accounting for1-6% of all neoplasms of the head and neck, and are diverse with respect to origin and pathology.[1] They are classified according to the 2005 World Health Organization, which lists 24 different histologic subtypes.[2] The most common histopathologic types are as follows: mucoepidermoid carcinoma (MEC), adenoid cystic carcinoma (ACC), adenocarcinoma not otherwise specified and salivary duct carcinoma. In general, they are typically divided into those arising from the major salivary glands and those arising from the minor salivary glands. Parotid glands is the most common site of major SGCs, followed by submandibular glands and sublingual glands. Also, minor salivary glands are the source of SGCs, representing for 9-23% of all salivary gland tumors.[3–5] Oral cavity is the most common site of minor SGCs, and hard palate is the most frequent subsite, as demonstrated in the previous studies.[3, 6–8] In contrast to major salivary gland tumors are almost benign, up to 80% of tumors arising from the minor salivary glands are malignant.[9, 10] Primary carcinomas originating from major salivary glands can be staged according to the 7th edition of the Union for International Cancer Control (UICC), whereas the stage of minor SGCs is mainly according to the primary site of the lesions.

Complete surgical resection, with adequate free margins, is currently the mainstay treatment for SGCs. Elective treatment of the N0 neck remains a controversial topic. Postoperative radiotherapy (PORT) can be used as an adjuvant therapy in patients with high-risk factors. And little is known about the efficacy of chemotherapy for advanced SGCs due to the rarity of the disease. It's still a great challenge to select effective therapeutic pathways for patients with recurrent tumors and those with unresectable or metastatic cancer.

In addition, very few clinical trials were designed to investigate the efficacy of novel treatment strategies. In the present review, the topics covered include surgery and radiotherapy, selective neck dissection, chemotherapy, and targeted therapy, which aimed to summarize the optimal management approaches and therapeutic outcomes of these disease and develop recommendations for management of malignancies in salivary gland.

## SYSTEMATIC LITERATURE REVIEW

We performed a systematic literature search via PubMed including articles in the English language. Medical subject headings and main keywords used in the PubMed were salivary gland, malignancy/carcinoma/cancer, management, elective neck dissection, chemotherapy, and radiotherapy. Relevant articles, abstracts, and review articles were selected and reviewed, we also scanned the references in the retrieved articles.

## SURGERY AND RADIOTHERAPY

In the past, various therapeutic means included surgery, radiotherapy (RT) and chemotherapy were used for SGCs, which mainly depended on the lesions’ specific situation, surgeons’ clinical judgment, and patients’ willingness to undergo resection. Up to now, controversy also exists as to whether RT alone, surgical treatment alone or combined surgery with RT is the optimal therapeutic approach. We have to rely on some studies to discuss about these relevant issues are as follows.

### Surgery combined with PORT *versus* RT alone

Most patients with early-stage lesions that are resectable generally tend to undergo surgery as their initial therapeutic approach, whereas those with advanced or unresectable cancers tend to be treated with RT alone or chemoradiotherapy (CRT), which hampered the comparison of the efficacy of RT alone with that of surgery combined with adjuvant RT. But some effort had been made to reflect the role of surgery in SGCs. For this part, PORT *vs*. RT alone studies are summarized in Table 1.

**Table 1:** PORT *vs*. RT alone

Study	Year	Neck treatment		Treatment	Median dosage	*n*	Survival rate	*P*
		RT	ND					
Liu et al. [11]	2008	9	-	S+RT RT	69.7 Gy 71.4Gy	10 10	54.8% (5-year OS) 0%	0.024
Cianchetti et al. [12]	2009	-	21	S+RT RT	69.6 Gy 74.3 Gy	76 64	55% (10-year OS) 35%	0.027
Mendenhall et al. [13]	2005	120	59	S+R RT	66 Gy 74.0Gy	160 64	48% (10-year OS) 35%	0.0482
Mendenhall et al. [14]	2004	55	13	S+RT RT	67.8 Gy 72.4 Gy	59 42	77% (5-year AS) 57%	NS
Terhaard et al. [15]	2005	120	-	S+RT	62.6 Gy 63 Gy	386 40	94% (5-year LC) 50%	<0.0005
Schramn et al. [16]	2001	-	15	S+RT	52-66 Gy	23	67% (5-year DFS)	NS
Iseli et al. [17]	2009	-	-	S+RT RT	62.0 Gy 66.0 Gy	93 10	75.5% (10-year LRFS) 24.6%	0.001

Abbreviation: n, the number of patients; RT, radiotherapy; S, surgery; OS, overall survival; DFS, disease-free survival; AS, absolute survival; LC, local control; LRFS: local recurrence-free survival; NS, not stated.

A retrospective study by Liu et al. revealed that the 5-year disease free survival (DFS) rate and overall survival (OS) in the surgical treatment group and the nonsurgical treatment group were 68.6% and 87.5% *vs*. 0% and 47.9%, respectively. Multivariate analysis indicated that surgical treatment was the only independent factor affecting DFS (*P* =.015), whereas surgical treatment (*P* = .024) was an independent factors affecting OS. [11] Cianchetti et al. carried out a retrospective analysis and included a series of 140 patients with minor SGCs to analyze the outcomes of patients receiving RT alone or combined RT with surgery. The median RT dose for patients receiving radical RT or PORT patients were 74.3 Gy (range, 50.0-79.2 Gy) and 69.6 Gy (range, 10.5-85 Gy), respectively. They found that patients receiving surgery plus RT had a higher 10-year local control (LC) rate (86% *vs*. 46%, respectively; *P* < 0.0001), 10-year local-regional control rate (76% *vs*. 44%, respectively; *P* = 0.0004), 10-year cause-specific survival rate (67% *vs*. 44%, respectively; *P* = 0.0295), 10-year OS rate (55% *vs*. 35%, respectively; *P* = 0.0277) than those receiving RT alone. Multivariate analysis also confirmed that treatment modality was a significant factor influencing patients’ survival (*P* =.0174). [12] Similar to the findings mentioned above, Mendenhall et al. [13] studied the treatment outcomes of 101 patients with head and neck ACC, and they found that the 5- and 10-year LC rates of the RT group and the surgery plus RT group were 56% and 43% *vs*. 94% and 91%, respectively; multivariate analysis of LC revealed that treatment group significantly influenced this endpoint (*P* = .0008). Moreover, the 5- and 10-year absolute survival rates of RT alone compared with surgery plus RT were 57% and 42% *vs*. 77% and 55%, respectively, which were consistent with the results of another study of Mendenhall et al. [14] Terhaard et al reported on 426 patients with minor salivary gland carcinomas treated with primary or postoperative radiotherapy in centers of the Dutch Head and Neck Oncology Cooperative Group. 386 patients were in PORT group with a median dose of 62 Gy. Primary radiotherapy (*n* = 40) was given for unresectable disease or M1, with a dose range of 28-74 Gy. The 5-year local control were 94% for PORT *vs*. 50% for primary radiotherapy. [15] Schramm and Imola study revealed that patients with locally advanced stage can also benefit from surgical treatment. They enrolled 23 patients presenting with T3-T4 lesion in the nasopharynx who received combined surgical resection with RT. The treatment outcomes were as follows: the 5- and 10-year DFS rates were 67% and 48%, respectively, and the 5-year local control rate was 77%. [16] Therefore, good survival outcomes might be achieved by combined extensive surgical treatment with RT for patients presenting with T3-T4 lesions.

Besides, the value of salvage surgery remains significant. Iseli et al. found that the 5-year survival rate was significantly better for locally recurrent patients who received salvage surgical treatment than who didn't (*P* = .006). The median survival time were 90.0 and 14.7 months, respectively. [17] Other studies also indicated that surgical treatment significantly influenced OS for patients presenting with recurrent disease (*P* < .0001). [18–20] In addition, primary RT is mainly reserved for patients with inoperable disease, those who refuse surgery or those who have an unresectable lesion.

### Surgery combined PORT *versus* surgery alone

Takes its unique clinical behavior into consideration, surgery does represent a potential treatment option, especially for ACC. Besides, it's also considered to be high-grade malignancy and often treated with combined-modality therapy. Available data comparing surgery alone with surgery plus PORT that used to verify the role of adjuvant RT had reflected in some retrospective studies. For this part, PORT *vs*. surgery alone studies are summarized in Table 2.

**Table 2:** PORT *vs*. surgery alone

Study	Year	Treatment	Median dosage	*N*	Survival rate	*P*	LC/RC rate	*p*
Armstrong et al. [22]*	1990	PORT S	56.6 Gy	46 46	51% (5-year DS) 10%	0.015	51.3% (5-year LC) 16.8%	0.14
Terhaard et al. [15]	2005	PORT S	62.6 Gy	386 112	NS		91% (10-year LC) 76%	0.0005
Storey et al. [23]	2001	PORT S	60.0 Gy	83 83	NS		88% (5-year LRC) 50%	<0.05
North et al. [24]	1990	PORT S	60.0 Gy	50 19	75% (5-year AS) 59%	0.014	NS (10-year LC)	<0.001
Le et al. [26]	1999	PORT	60.0 Gy	52	63% (10-year OS)	NS	88% (10-year LC)	NS
Terhaard et al. [27]#	2003	PORT S	62.0 Gy	385 113	NS	NS	89%( 10-year RC) 67%	0.03

Abbreviation: n, the number of patients; S, surgery; PORT, postoperative radiotherapy; LC, local control; RC, regional control; LRC, locoregional control; DS, determinate survival; AS, actuarial survival; NS: not stated; OS, overall survival; *, for patients with III and IV disease; #, for N+ patient

Terhaard et al. in a large retrospective study enrolled 498 patients presenting with malignant SGCs who received surgery follow by RT (*n* = 398) or surgery alone (*n* = 112). Despite a greater frequency of poor prognostic features in the radiation therapy group, such as more positive neck nodes, more locally-advanced tumors, they also revealed that the relative risk of local recurrence in patients receiving surgery alone was 9.7-fold that of patients receiving combined surgery with RT. And the 5- and 10- years of actuarial local control rates were significantly higher for combined surgery with RT (94% *vs*. 84% for surgery alone, 91% *vs*. 76% for surgery alone) (*P* = 0.0005). In addition, PORT significantly improved 10-year local control rate compared with surgery alone in patients with high-risk factors for SGCs, such as T3-4 tumors (84% *vs*. 18%, *p* < 0.001), close resection margins (95% *vs*. 55%, *P* = 0.003), incomplete resection (82% *vs*. 44%, *p* < 0.001), bone invasion confirmed by pathologic (86% *vs*. 54%, *P* = 0.04), and perineural invasion (88% *vs*. 60%, *P* = 0.01). Also, PORT significantly improved regional control compare with surgery alone in the pN+ neck (86% *vs*. 62%, *P* = 0.03).[15]. Zeidan et al. used the SEER database to investigate the role of adjuvant RT in minor SGCs. Multivariate Cox analysis showed adjuvant RT correlated with a 24% survival advantage as compared to surgery alone (HR 0.76, *p* = 0.02). And advanced T/N category, adenoid cystic histology, high grade, and nasal cavity/paranasal sinus location were also associated with decreased survival. [21]

A matched-pair analysis conducted by Armstrong et al. included 46 patients with previously untreated, non-metastatic malignancies of major salivary gland origin who received combined surgery and RT between 1966 and 1982, compared with 46 patients treated with surgery only between 1939 and 1965, who were matched according to prognostic criteria. They found that the 5-year determinate survival and 5-year local control for stage III (Tl-2, N1, M0) or IV (T3-4, N1, M0 or T4, N0, M0) disease in patients who received combined-modality therapy versus patients who underwent surgery alone were 51% *vs*. 10% (*P* = .015) and 51% *vs*. 17% (*P* = .014), while no significance was found in 5-year determinate survival for all patients and stage I and II patients. For patients with nodal metastases, the 5-year determinate survival for PORT and surgery alone was 49% *vs*. 19% (*P* = .015). The corresponding 5-year local-regional control rate was 69% *vs*. 40% (*P* = .05).[22] There are some other studies have showed that PORT for head and neck SGCs improves locoregional control and long-term survival in patients with locally advanced disease, high-grade (poor-differentiated) tumors, positive margins, perineural invasion, or positive lymph nodes.[23, 24] Storey et al. revealed that adenocarcinoma, high-grade histology were associated with decreased locoregional control and DFS, and high-risk patients presented with submandibular gland lesions received combined surgery and PORT had a better 10-year actuarial locoregional control rate (88% in the current study *vs*. 50% for surgery alone in previous studies). [23] Le et al. also indicated that patients with adenocarcinoma histology, and sinonasal and oropharyngeal primary sites were associated with worse local control. [26]

However, for the early stage (I and II) disease, some authors demonstrated that the addition of PORT didn't bring survival benefit. A study conducted by Terhaard et al. revealed that the addition of PORT hasn't improved the 10-year local control, compared with patients received surgery alone in T1 (95% *vs*. 83%, *P* = not significant) or T2 (91% *vs*. 88%, *P* = not significant) lesion. Furthermore, for patients with negative resection margin, 10-years local control rate was 98% for patients with PORT *vs*. 90% with surgery alone (*P* = not significant).[15] Armstrong et al. also found that no significantly better combined with PORT in 5-year determinate survival for stage I and II patients (81.9% *vs*.95.8% for surgery alone; *P* =not significant).[22] Therefore, some scholars considered that PORT can be omitted without loss of disease control only when early-stage (stages I and II) patients with clear margin, and without adverse prognostic factors such as lymphovascular or perineural invasion that is a set of conditions usually restricted to low-grade variants.[25–28]

In summary, surgery predominates the treatment for SGCs, and PORT was recommended in patients with adverse prognostic factors, such as T3 or T4 tumors, close or incomplete resection margins, high grade, perineural or vascular invasion, and positive lymph nodes. While, the role of adjuvant RT for T1 or T2 patients with complete resection hasn't been confirmed. Primary radiation therapy, particularly, fast neutron radiotherapy appears to be the treatment of choice for patients with inoperable tumors or those with other comorbidities. Thus, for the most part, patients treated with primary radiotherapy had an unfavorabe prognosis. Due to the lack of randomized prospective trials, the benefit of adjuvant RT has never been demonstrated.[29, 30] Therefore, Large-scale, prospective clinical trials should be conducted to verify the value of adjuvant RT.

## ELECTIVE NECK DISSECTION

Cervical lymph node status is an important prognostic predictor for SGCs. [18–20] Recent and past studies are consistent in revealing a reduced survival in patients with positive lymph node at time of primary therapy, and the 5-year survival rate was significant different with or without cervical lymph node metastasis (44-48 % *vs*. 73-77 %). [20, 31–34]Therefore, management of the cervical lymph nodes warrants particular discussion. For patients with clinically positive cervical lymph node, therapeutic neck dissection (TND) is still strongly recommended at the time of primary surgery followed by adjuvant RT, regardless of histology or site.[35, 36] However, elective treatment of the clinical N0 neck remains a controversial topic. And treatment of the clinically negative neck included observation, elective ND, and prophylactic radiation.

The incidence of occult lymph node metastasis from SGC has generally been reported to range from10% to 20%. [37, 38] Elective neck dissection (END) should be indicated when the risk of subclinical disease in a clinically negative neck exceeds 15%.[36] Hence, it would be of great value to formulate a criteria to select patients for whom a neck dissection should be incorporated into the surgical management of the primary tumor.

Intuitively, the histology type of the primary tumor should be an important factor in the risk of occult metastasis. Over the past decades, several studies have shown that certain tumor pathologies carry a powerful significant trend in the risk of occult nodal involvement. A large retrospective study conducted by Lloyd et al. enrolled a total of 2667 patients of minor SGCs and found that 426 (16.0%) of cases had neck nodal involvement. They revealed that histologic grade was a significant predictor of nodal metastasis for MEC or adenocarcinoma but not for ACC.[39] As is known to all, certain histological type, such as ACC, is associated with a low rate of lymph node metastasis. [23, 40] Spiro et al. ever reported the lymph node metastasis rate about ACC in the major and minor salivary glands was 7.4% on initial presentation. [31] Jenkins et al. and Spiro et al. revealed that elective surgical treatment was recommended for patients with high-grade cancers such as high-grade MEC or high-grade adenocarcinoma.[41, 42]

In general, high-grade tumors are more frequently associated with occult metastasis than are low-grade tumors. A landmark study was published by Armstrong et al. in which the lymph node metastasis rates were retrospectively assessed in 474 SGCs. 47 of 407 patients (12%) had occult nodal metastasis (i.e. clinically negative, while pathologically positive lymphadenopathy). Multivariate analysis revealed that tumor grade has a statistical significance with occult metastases.[43] And the authors were recommended that END should be only applied for high grade tumors (regardless of histologic type). A recent study also indicated that primary tumor histologic type and tumor grade were statistically significant predictors of occult lymph node disease. The cervical metastases rates of low-, intermediate-, and high-grade tumors were 0%, 10%, and 35%, respectively. [44] Schramm and Imola also found a 47% rate of occult metastasis in clinically node-negative necks (Eight of 14 had poorly-differentiated tumors). [16] Due to relatively high rate of occult neck involvement, Liu et al. also indicated that elective neck treatment included neck resection and radiotherapy was also recommended for patients with cervical negative lymph node, especially for high grade tumors. [45]

More notably, advanced tumor (T) stage is regarded as another important risk factor for occult disease. Armstrong et al. [43] found that among patients with N0 necks, T4lesions had a 24% risk of occult neck involvement and 16% for T3 lesions, versus 7% for T1/T2 lesions. They also showed an independent increased risk for primary tumors larger than 4 cm (20%) compared with those smaller than 4 cm (4%) (*P* < 0.0001). Neck treatment was only recommended for high-grade and larger tumors. Lloyd et al. had also revealed advanced (T3-T4) stage was significantly related to lymph node involvement. [39]

Most recently, some scholars proposed that site of primary tumor was a prognostic index of lymph node involvement for SGCs.[15, 39, 46] Site of the primary tumor (oral cavity 9%, parotid gland 25%, submandibular gland 42%, other locations 36%; *p* < 0.0001) was independent prognostic factors for the presence of positive nodes, as shown by Terhaard et al [15]. The authors recommended that elective treatment of the neck nodes is indicated for almost all submandibular tumors, except for T1 acinic or T1 adenoid cystic tumors. Elective treatment of the neck for tumors of the oral cavity is seldom indicated. Lloyd et al [39] found that pharyngeal site of primary involvement as predictive of lymph node metastasis for minor SGCs. In some sites such as the sinuses and nasal cavity, tumors can attain large sizes before they present clinically. Liu et al. [46] revealed that primary tumor sites which were located in the submandibular gland (40%), followed by the buccal mucosa (38.9%) were associated with the incidences of cervical metastases. Also, tumor size (>4 cm) was recognized as a poor prognostic factor for occult disease [39, 43]. Parotid tumors with facial paralysis are associated with a high percentage of occult lymph node metastases. [47, 48]

To conclude, a number of evidence suggests that TND should be recommended to those who has clinical or radiologic evidence of cervical node metastasis. While, therapeutic ND could be bring benefit to patients with advanced T stage, large tumors, or high-grade tumors in clinical N0 neck, especially for MEC and adenocarcinoma. In addition, in terms of the location of tumors, all other locations except the oral cavity should take TND into account.

## CHEMOTHERAPY

As mentioned above, surgery and/or radiotherapy are reserved to treat localized disease, while systemic therapy is of necessity to manage recurrent and/or metastatic SGCs. SGCs are characterized by rather frequent local recurrence and distant metastasis, and no satisfactory method of therapy has been reported. Very few clinical trials were designed to investigate the efficacy of systemic therapy, because of the rarity of the disease. Chemotherapy, which plays an important role in systemic therapy, is generally reserved for the palliative treatment of symptomatic locally recurrent and/or metastatic disease that is not amenable to further surgery or radiation. However, there are no National Comprehensive Cancer Network (NCCN) recommendations concerning specific chemotherapy regimens. Therefore, the treatment of recurrent and/or metastatic patients becomes a challenge. Conventional chemotherapy regimens, such as cisplatin and 5-FU or CAP (cisplatin, doxorubicin, and cyclophosphamide) are still utilized as first-line therapy for patients with advanced lesions. With various agents to be tested, only a few were considered to be effective, such as 5-fluorouracil, doxorubicin, and Cisplatin.[49–53]

Previous studies revealed that the value of chemotherapy has been proven to be limited to deal with the patients with recurrent or metastatic disease and no chemotherapy regimen has been the effort to prolong or improve OS or DFS in these tumors. In a retrospective study, various treatment agents included the chemotherapy or targeted drugs, such as adriamycin, cisplatin, carboplatin, 5-fluorouracil, methotrexate, docetaxel, bacilli Calmette Guerin, were offered to manage the majority of patients with symptomatic metastatic disease. However, the survival rates of patients with distant metastasis were similar with or without chemotherapy (35.2% *vs*. 27.6%, *P* = 0.747).[25] A review of systemic therapy in the management of recurrent or metastatic SGCs by Lagha et al. showed that the most effective chemotherapeutic agents seem to be platinum, 5-Fluorouracil and anthracyclines, and recommended that symptomatic locally recurrent or metastatic patients adapt platinum combined with doxorubicin to maximize the likelihood of a response, and for cases of slow disease progression and asymptomatic patients, a single agent therapy is sufficient.[54] However, another review revealed that for the cases of slow disease progression and asymptomatic patients, which often occurs in ACC, chemotherapy could be delayed until an evident progression of the disease or to the emergence of symptoms.[55] For single-agent therapy, either cisplatin or paclitaxel isn't recommended for recurrent and/or metastatic SGCs, on account of lack of availability of less toxic choices (cisplatin) and proven activity (paclitaxel). Whereas a cisplatin-based polychemotherapy for candidate patients may achieve a higher response rate (RR), compared with monochemotherapy (25 *vs*. 13%).[56] A meta-analysis, which enrolled about 200 patients with SGCs entered mainly into small Phase II trials, was made to reflect the role of chemotherapy and identified that platinum-based chemotherapy as an independent predictor of increased survival. Chemotherapy in most of these studies elected to the meta-analysis was done as a palliative intent, but the median survival has a significant increased with both platinum-based (2.5 months) and anthracic line-based (4.9 months) chemotherapy.[57]

Therefore, whether chemotherapy combined with PORT can improve OS and DFS or not? On this issue, some scholars had also made some effort to prove the efficiency of postoperative chemoradiotherapy (POCRT). Cisplatin-based therapy was the most common drugs given in combination chemoradiorherapy (CRT). Tanvetyanonet al. performed a case match comparison of POCRT versus PORT, with 12 cases in eachstudy cohort. 11 patients received a platinum-based regimen (8 cisplatin and 3 carboplatin), and only one patient received combined cisplatin and fluorouracil chemotherapy. Although grade 3 or higher toxicity (hematologic) was only seen in patients in the CRT cohort (*n* = 8), in no case was the toxicity severe enough to cause treatment cessation. The outcome revealed that the 3-year survival rate was 83% in the CRT group compare with 44% in the radiation-alone group (*P* =.05). [58] Recent studies have also indicated that POCRT, particularly with platinum-based chemotherapy, showed a trend toward higher locoregional control rates than those treated with PORT alone. [59–62] However, because of increased toxicity and mortality with the application of POCRT, some investigators did not recommend the use of POCRT for patients with SGCs. Tanvetyanon et al. conducted another retrospective study to compare adjuvant a platinum-based CRT with adjuvant radiotherapy among the older patient population. They found that treatment with adjuvant CRT was associated with an increased mortality and toxicity when compared to adjuvant radiotherapy.[63] Similarly to Tanvetyanon and the colleagues, Amini et al [64] retrospectively reviewed 2210 patients with resected major salivary gland carcinoma on the basis of the date from the National Cancer Data Base. They found that OS was significantly inferior with adjuvant CRT (*n* = 368) compared with RT alone (*n* = 1842) (*p* = .02), and patients with multiagent chemotherapy, CRT *vs*. RT alone appeared to have worse OS, compared with single-agent chemotherapy (*P* = .03). Therefore, the Radiation Therapy Oncology Group (RTOG 1008) conducted a phase II randomized trial to explore the utility of a platinum-based adjuvant CRT in high-risk patients. Univariate analysis revealed that the CRT group was identified to have inferior 3-year PFS (42.1% *vs*. 73.8%; *p* < .001), 3-year OS (52.2% *vs*. 78.1%; *p* = .004), locoregional control (79.3%*vs* 91.2%; *p* = .031), and distant metastasis-free survival(52.7% *vs*. 83.3%; *p* < .001) rates. Multivariate analysis revealed that there was a trend toward a benefit to PFS from CRT, but it was not statistically significant (*p* = .482).RTOG 1008 came to a conclusion that the standard use of CRT for high-risk salivary malignancies cannot be recommended. [65]

In summary, chemotherapy as a palliative treatment was applied to patients experiencing with symptomatic locally recurrent and/or metastatic disease which were not amenable to further surgery or radiation. A platinum-based chemotherapy regimen could be bring benefit to patients with incurable SGCs, especially for symptomatic or rapidly progressive patients. Future studies are required to identify new chemotherapeutic agents in order to improve the prognosis in patients with SGCs.

## TARGETED THERAPY

Due to the poor results with chemotherapy, it's urgent to explore novel therapeutic interventions for this disease. And great expectations have been put into individualized therapies: in particular, the EGF receptors family (EGFR and HER2), KIT and androgen receptors are the most commonly investigated molecular targets in SGCs. Their expression seems not to be linked to its pathogenetic role in the development of SGCs, but more to the histogenetic origin of the tumor cells. Various targeted agents, such as imatinib, cetuximab, gefitinib, trastuzumab, had been used for exploring new treatment for SGCs, but on account of the rare incidence of SGCs, the number of cases available on targeted therapy for analysis is relatively small. The following we described was about the targeted therapy of SGCs.

According to the literature reported, high expression of c-kit has been noted in up to 90% of salivary ACC.[66–70]To this day, the relationship between gene mutation and the mechanism of c-kit activation hasn't been clearly identified in this tumor.[66–70] Owing to the rare incidence of salivary ACC, a total of eight studies have evaluated imatinib in over 80 patients with advanced ACC (7 studies have used imatinib alone, and one study has evaluated imatinib combination with cisplatin), only 4 partial response, with an objective response rate of 5% and a short response duration (range, 9-15 months).[71–78]

Likewise, it is well established that epidermal growth factor receptor (EGFR) is frequently positive in salivary ACC (74-91%). So, the EGFR antibody, such as cetuximab, seemed promising. Some effort had been made to evaluate the value of cetuximab. A phase II trial conducted by Hitre et al. revealed that the combination of cetuximab with cisplatin for patients with metastatic ACC presented an objective response rate>40% and median PFS and OS were 13 and 24 months, respectively. [77] With respect to the outcome, which has improved treatment efficacy compare with those without cetuximab, and side effects were also manageable and endurable.[79] Other EGFR antibody, such as gefitinib, lapatinib, were also used in the treatment of metastatic salivary gland cancer, particularly in ACC. However, therapeutic outcomes were disappointing and failed to achieve any objective responses.[80, 81] Therefore, the antitumor activity of EGFR antibody in metastatic and/or recurrent SGCs still needs to explore further.

Bortezomib, a proteosome inhibitor, which was the first one and the only one to approved to enter clinical. It was also investigated in patients with metastatic ACC to assess its effectiveness. Argiris et al. conducted a phase II trial which enrolled 25 patients with advanced ACC to evaluate the activity of bortezomib. Side effect was well tolerated and did not hamper the treatment course, but no complete or partial responses was found from bortezomib in monotherapy, 15of 21 evaluable patients (71%) presenting with best response was stable disease for a median duration of 4.2 months (range: 0-20.1 months).[82]

Up to date, none of the targeted therapies aforementioned have shown any real antitumor activity in SGCs. The best response obtained was PR in a small group of patients, and only a short period of time (a few months). Currently, several trials on targeted therapy involving SGCs are still ongoing.

In addition, other therapy such as hormone therapy has been reported in the literature. Several studies showed that some SGCs possess hormonal receptors, such as estrogen or progesterone or even androgen receptors in salivary duct carcinoma.[83–87]

So far, no phase II studies have been carried out, and the effectiveness of hormone therapy is still a mystery. Therefore, it is very difficult to define the role of hormone therapy in SGCs.

## CONCLUSION

Salivary gland malignancies as a heterogeneous group have a relatively low of incidence, but a variety of histological types. And their rarity limits study size and the ability to perform phase III trials. The current therapies available for the management of patients with SGCs is complete surgical resection, which is the mainstay treatment for these lesions. At the same time, therapeutic ND should be recommended to those who has clinical or radiologic evidence of cervical node metastasis. While, therapeutic ND could be bring benefit to patients with advanced T stage or high-grade tumors in clinical N0 neck, especially for MEC and adenocarcinoma. For patients with inoperable disease, those who refuse surgery or those who have an unresectable tumor, primary RT should be considered. And PORT was recommended in patients presenting with adverse prognostic factors, such as T3-4 tumors, close or incomplete resection margins, high grade, perineural or vascular invasion, and positive lymph nodes. While, the role of adjuvant RT for T1 or T2 patients with complete resection, and the value of targeted therapies for advanced and/or metastatic patients have never been confirmed. Chemotherapy as palliative treatment to deal with the patients with recurrent and/or metastatic disease, which has been proven to be of limited effect, and no chemotherapy regimen has been the effort to prolong or improve OS or DFS in these tumors. Meanwhile, the results of targeted therapy have been disappointing, especially the objective responses reported in several studies. Large-scale, prospective clinical trials or phase II trials should be conducted to prove the role of adjuvant treatment, such as chemotherapy, targeted therapy, hormone therapy.

Overall, a broad spectrum of choices exists for the management of SGCs, with surgical treatment at the center for most therapeutic plans. At present, further clinical trials based on collaborative multicentric efforts should be conducted to establish new treatment guidelines for these patients.
